# Beyond Angiography: Intracoronary Imaging Revealing a Left Anterior Descending (LAD) Clot in a 45-Year-Old Male Patient

**DOI:** 10.7759/cureus.96084

**Published:** 2025-11-04

**Authors:** Ayobami B Omodara, Seena Darwin, John Fahy, Habib Rahman, Shah R Mohdnazri

**Affiliations:** 1 Cardiology, Southend University Hospital, Mid and South Essex NHS Foundation Trust, Southend-on-Sea, GBR; 2 Internal Medicine, Manchester University Hospitals NHS Foundation Trust, Manchester, GBR; 3 Cardiology, Southend University Hospital, Southend-on-Sea, GBR; 4 Interventional Cardiology, Essex Cardiothoracic Centre, Mid and South Essex NHS Foundation Trust, Basildon, GBR

**Keywords:** cardiology imaging, clinical cardiology, internal medicine (general medicine), non-st segment elevation myocardial infarction (nstemi), oct (optical coherence tomography)

## Abstract

Intravascular imaging has become an invaluable adjunct in the evaluation of ambiguous coronary lesions, particularly when conventional angiography is inconclusive. We report the case of a previously healthy 45-year-old man who presented with acute chest pain and elevated dynamic troponin levels, but without ischemic changes on electrocardiography. His initial invasive coronary angiography revealed a filling defect in the proximal left anterior descending (LAD) artery, which raised the suspicion of an intracoronary thrombus.

To further characterize the lesion, intravascular ultrasound (IVUS) and optical coherence imaging (OCT) were performed. Optical coherence tomography (OCT) was performed for clarification and demonstrated a ruptured atherosclerotic plaque with surface irregularity, accompanied by a secondary red thrombus (a thrombus rich in red blood cells and fibrin), as opposed to an isolated primary LAD thrombus. This finding directly influenced the treatment strategy and enabled optimized stent apposition, expansion, and deployment.

The case underscores the value of OCT in resolving diagnostic uncertainty when angiographic appearances are equivocal, ensuring an accurate diagnosis and guiding appropriate intervention. In addition to this, OCT-guided percutaneous coronary intervention also markedly improves accuracy in stent deployment, ensuring optimal patient outcomes.

## Introduction

Coronary angiography remains the gold standard for evaluating coronary artery disease; however, its two-dimensional, luminographic nature can sometimes limit diagnostic accuracy, particularly in assessing ambiguous or complex lesions. Intravascular imaging modalities, such as intravascular ultrasound (IVUS) and optical coherence tomography (OCT), have emerged as indispensable tools, providing high-resolution, cross-sectional visualization of the coronary vessel wall, plaque morphology, and stent-vessel interactions.

OCT, with its near-microscopic resolution, enables the precise characterization of plaque components, the detection of plaque rupture or erosion, the identification of intracoronary thrombus, and the assessment of stent expansion and apposition [1]. These detailed insights can significantly influence clinical decision-making, particularly in cases where angiographic findings are equivocal or potentially misleading [2].

Acute coronary syndrome (ACS), encompassing unstable angina, non-ST elevation ACS (NSTE-ACS), and ST-elevation ACS (STEMI/STE-ACS), typically presents with chest pain, with or without electrocardiographic changes and troponin elevation [3]. It is uncommon to encounter acute coronary thrombus in the absence of plaque disruption or underlying atherosclerotic cardiovascular disease (ASCVD). In this case, the patient exhibited two core symptoms of ACS, but invasive angiography revealed an ambiguous filling defect. It was therefore crucial to exclude a thrombus in the left anterior descending (LAD) artery, as its presence would alter management strategies, including consideration of anticoagulation and evaluation for potential paroxysmal tachyarrhythmias such as atrial fibrillation.

Isolated LAD artery thrombosis represents a rare but clinically significant form of ACS, often resulting in extensive myocardial infarction (MI) due to the LAD's critical role in supplying the anterior left ventricular (LV) wall [4]. In contrast, plaque rupture, a more common etiology of ACS, typically involves the disruption of a vulnerable atherosclerotic plaque, exposing thrombogenic material and triggering thrombus formation, which is generally managed via percutaneous coronary intervention (PCI) according to established guidelines [5]. While both conditions share some initial management strategies, LAD thrombosis may necessitate more aggressive or tailored interventions due to a higher thrombus burden and increased risk of distal embolization. This consideration guided the decision to perform PCI under OCT guidance, as discussed in this report.

We present the case of a 45-year-old previously healthy man who presented with acute chest pain and elevated cardiac biomarkers. Coronary angiography revealed an ambiguous filling defect in the proximal LAD, initially suggestive of intracoronary thrombus. Subsequent OCT imaging provided definitive clarification, identifying a ruptured atherosclerotic plaque with superimposed red thrombus. This case highlights the critical diagnostic and therapeutic value of OCT in guiding optimal management for patients with uncertain angiographic findings.

## Case presentation

A 45-year-old man presented to the accident and emergency department with left-sided, dull, aching chest pain rated 7/10 in severity, associated with heaviness radiating to the left arm and neck. This was accompanied by nausea, diaphoresis, and dizziness.

He first experienced these symptoms the day prior while mountain biking immediately after intense exertion; the episode resolved spontaneously after three to four hours. He did not seek any medical attention as he felt it was the flaring up of his left shoulder injury. He also reported intermittent exertional palpitations, relieved by rest, with his Apple Watch recording a heart rate between 180 and 195 bpm during the episode the previous day. The following day, the chest heaviness recurred while he was driving his work van. He stopped driving, called emergency services, and was administered aspirin 300 mg orally (stat) and sublingual glyceryl trinitrate spray by paramedics, which did not completely alleviate his symptoms. The chest pain persisted for over an hour.

The patient is otherwise independent and highly physically active. He lives with his partner and child, works in the construction sector, and rides motorcycles recreationally. He is an ex-smoker with a 20 pack-year history (10-15 cigarettes/day for 29 years), having quit five years ago; he currently vapes. He denies alcohol consumption above recommended limits. He reports no exertional dyspnea, orthopnea, paroxysmal nocturnal dyspnea, or peripheral edema. There is no family history of premature coronary artery disease or ASCVD. He has no personal history of angina and reports good exercise tolerance.

On examination, he was alert and pain-free. He had a good radial pulse bilaterally, with a rate of 88 beats/minute, blood pressure of 124/76 mmHg, and an oxygen saturation of 98% on room air. Heart sound was normal without any murmurs, and the chest sounded clear bilaterally. There was no pedal edema, and his jugular venous pulse was not visible. Other general physical examinations were normal.

A blood test revealed that Troponin T was elevated to 217 ng/L from an initial reading of 159 ng/L, recorded one and a half hours prior. D-Dimer was negative. The patient's laboratory results are as follows: Hemoglobin levels were recorded at 162 g/L, with a white blood cell count of 15.5 × 109/L and a platelet count of 342 × 109/L. Renal function and electrolyte levels were within normal limits, indicated by a satisfactory estimated glomerular filtration rate. Additionally, the lactate level was measured at 0.6, the C-reactive protein level was 1, and the international normalized ratio was determined to be 0.9 (see Table 1).

**Table 1:** Blood results Initial blood result shows dynamically raised hs troponin levels INR: international normalized ratio; aPTT: activated partial thromboplastin time; hs: high sensitivity

Parameters	Results	Range	Unit
Hemoglobin	162	115-165	g/L
White cell count	15.5	4.0-11.0	×10^9^/L
Platelet count	342	150-400	×10^9^/L
Neutrophil count	12.15	1.7-7.5	×10^9^/L
Sodium	137	133-146	mmol/L
Potassium	4.1	3.5-5.3	mmol/L
Urea	7.8	2.5-7.8	mmol/L
Creatinine	88	45-85	umol/L
INR	0.9	0.8-1.2	INR
Prothrombin time	10.6	10.3-13.3	seconds
APTT	26.2	25.7-35.3	seconds
First hs troponin T	159	<14	ng/L
Second hs troponin T	217	<14	ng/L
C-reactive protein	1	<5	mg/L

**Figure 1:**
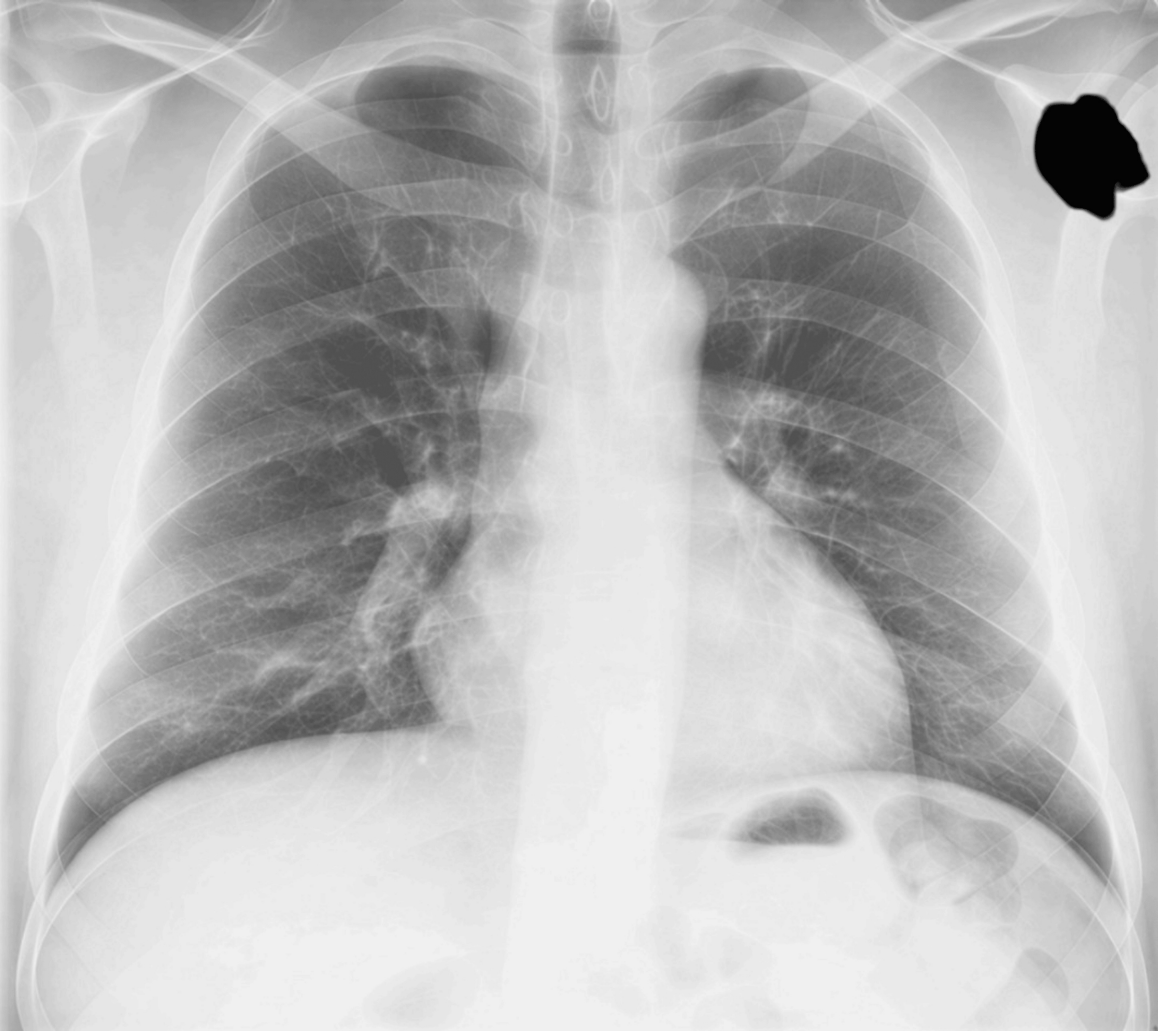
Chest X-ray with normal cardiac silhouette and no features of congestion or consolidation Black mark is used to ensure complete anonymity

His chest X-ray appeared normal with a normal cardiac silhouette and no features of pulmonary vascular congestion or consolidation (Figure 1).

**Figure 2:**
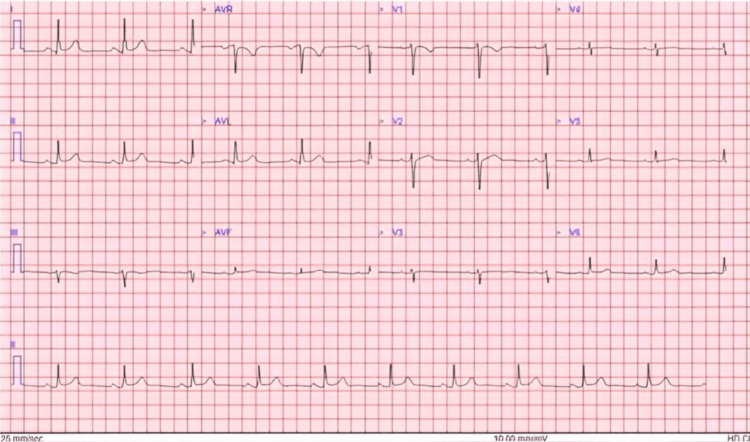
The ECG upon admission to the coronary care unit was largely unremarkable and very similar to the ECG recorded by the ambulance crew ECG: electrocardiogram; AVR: augmented vector right; AVL: augmented vector left; AVR: augmented vector foot

An initial electrocardiogram (ECG) was nonspecific with widespread subtle ST elevation but no obvious PR depression. There was no prior ECG to compare. No obvious acute ischemic pattern was identified on subsequent serial ECGs (see Figure 2).

He was loaded on full NSTE-ACS therapy, which included dual antiplatelet therapy (aspirin 300 mg + ticagrelor 180 mg), a proton pump inhibitor (omeprazole 20 mg once daily), bisoprolol 2.5 mg once daily, and a high-dose statin (80 mg atorvastatin) every night. His bedside echocardiogram showed normal LV structure and function without evidence of acute MI complications, and all valves were intact. A more formal echocardiogram was performed, which showed normal systolic and diastolic function with an LV ejection fraction of more than 55% and normal LV size. There were no regional wall motion abnormalities or hypokinetic regions. The valves were normal throughout the study.

**Figure 3:**
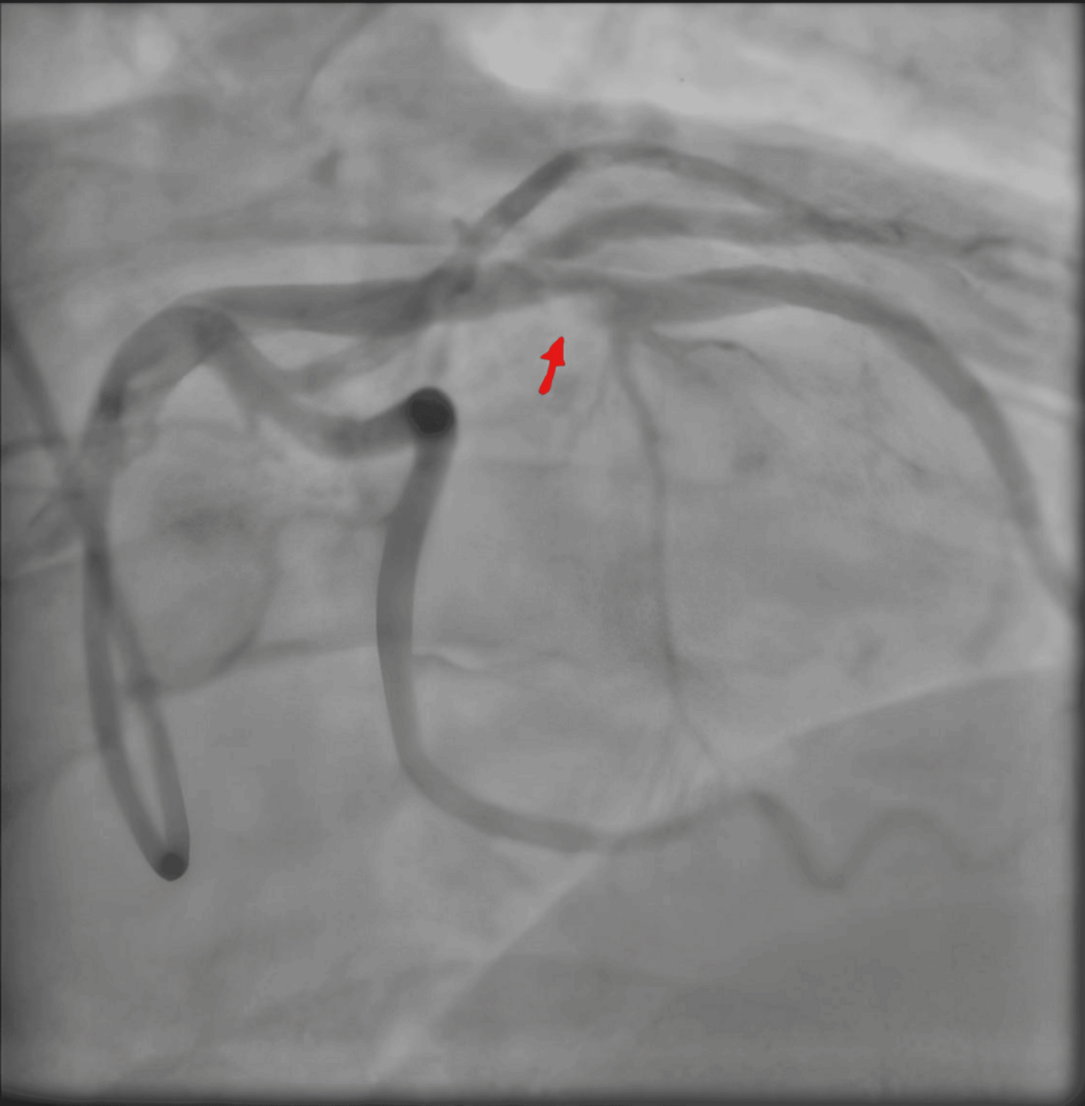
Inpatient invasive diagnostic coronary angiogram The red arrow shows subtotal mid-LAD artery opacification projected as a finger-like filling defect that raised the suspicion of a mid-LAD thrombus or classic plaque rupture LAD: left anterior descending

**Figure 4:**
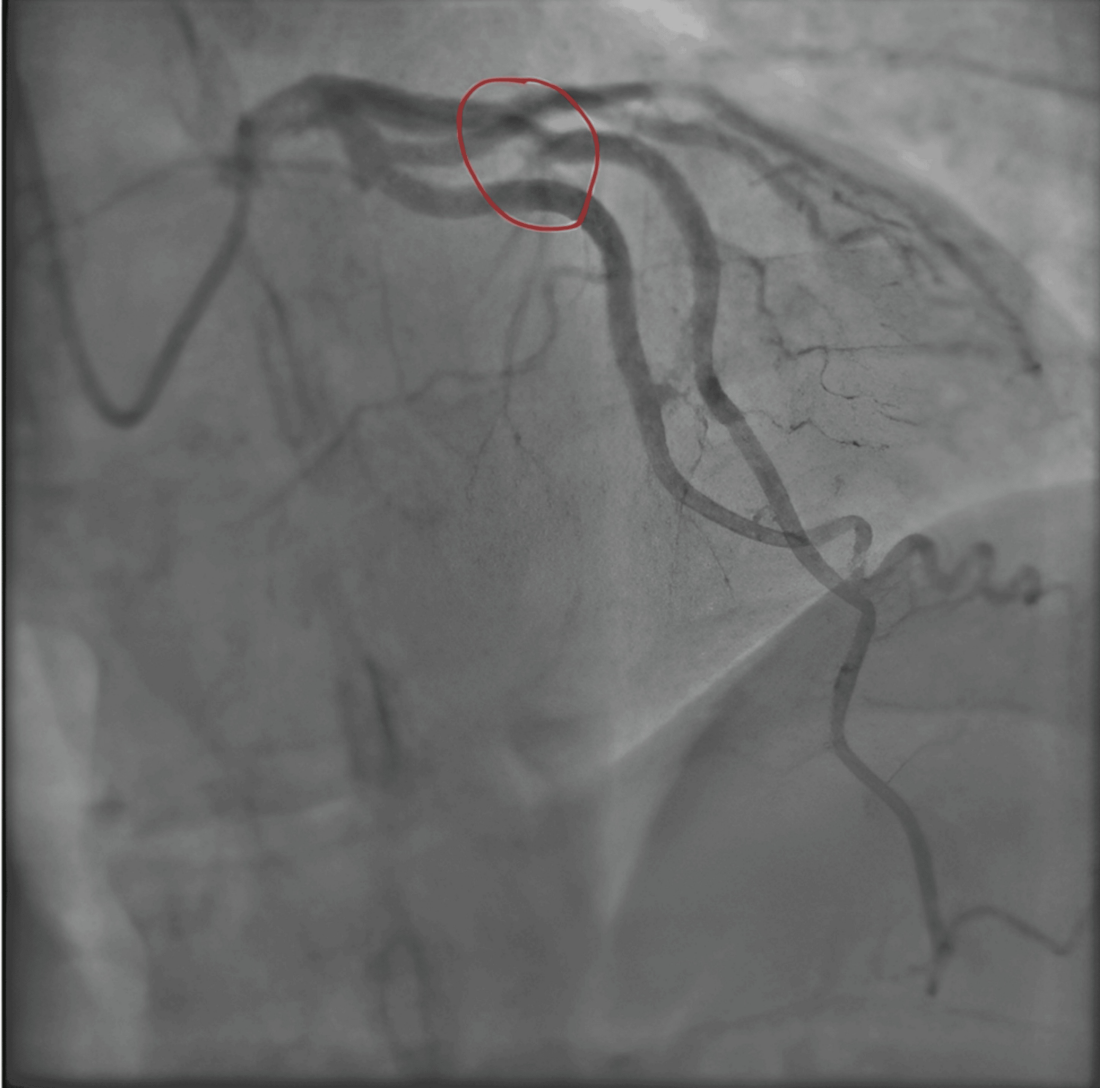
Different view of the LAD coronary artery Red circle demonstrates subtotal occlusion in the proximal to mid vessel LAD: left anterior descending

He was then transferred to the coronary care unit on telemetry. He remained stable on telemetry and had an inpatient invasive diagnostic coronary angiogram the next day (see Figures 3, 4).

**Figure 5:**
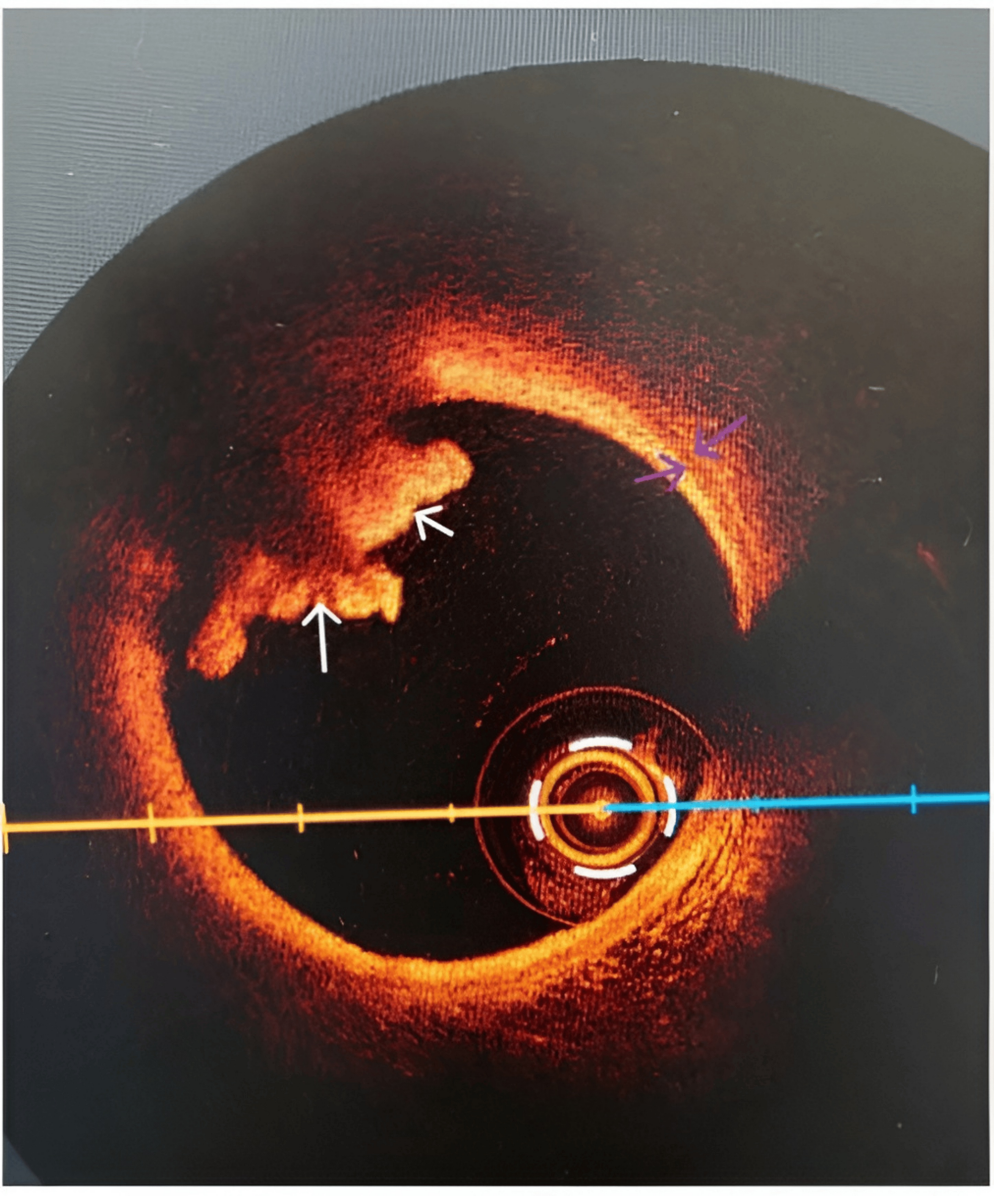
OCT image depicting the red thrombus (white arrows) and ruptured plaque at the base of it Analyzing cross-sectionally, the inner (left) purple arrow depicts the IEL layer, the outer (right) purple arrow shows the EEL layer, and the structure in between is the tunica media. Clear EEL visualization helps determine the true vessel boundaries, ensuring appropriate stent diameter selection, adequate lesion coverage, and optimal stent expansion while minimizing the risks of malapposition or vessel injury Red thrombus: rich in red blood cells and fibrin with high backscattering and strong signal attenuation as opposed to a white thrombus, typically rich in fibrin and platelets, homogeneous with low backscattering and little signal attenuation (not represented in the above image) IEL: internal elastic laminate; EEL: external elastic laminate; OCT: optical coherence tomography

**Figure 6:**
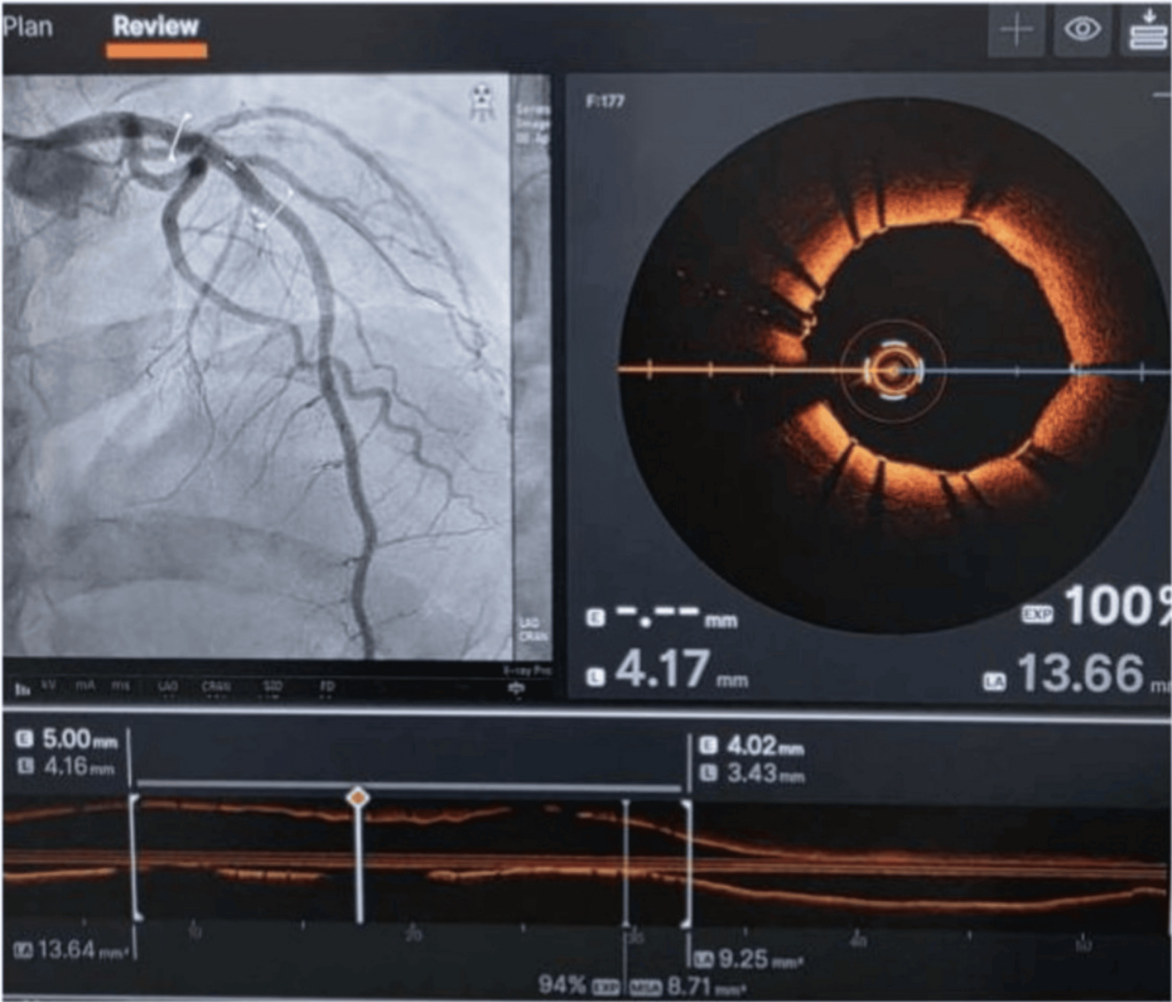
Images after insertion of stent Stent apposition in the image on the right; image on the left shows direct correlation with invasive angiogram in the mid-segment (white dash); the bottom image, white pin with orange head, shows the longitudinal section of the lumen with the stent affixed

**Figure 7:**
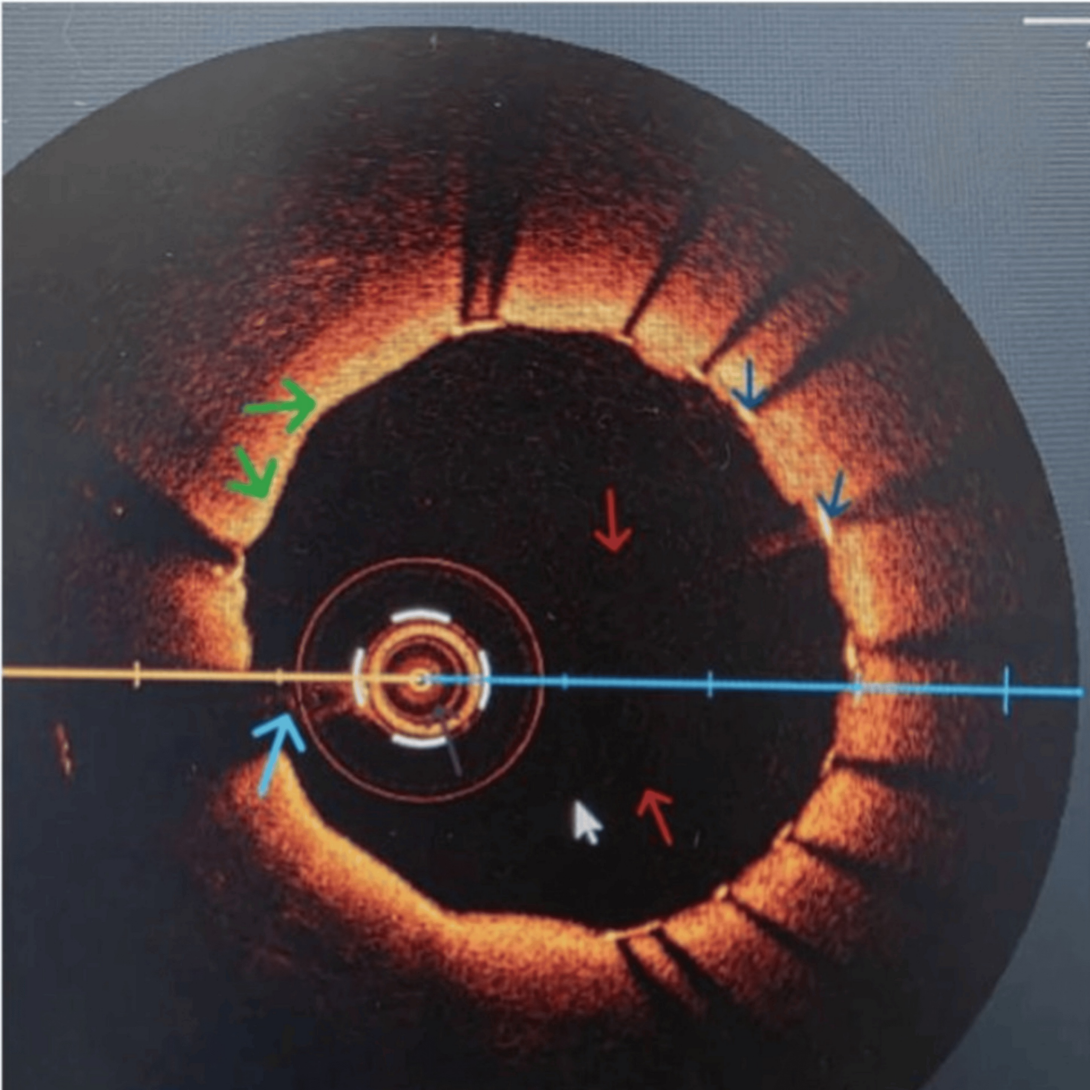
Another OCT image after stent insertion Dark blue arrows depict a stent with deflected comet-like shadows; red arrows show IVUS lumen devoid of blood; green arrows depict a ballooned lesion now buried within the intima with the DES, and the light blue arrow shows the guide wire DES: drug-eluting stent; OCT: optical coherence tomography; IVUS: intravascular ultrasound

His diagnostic angiogram, as seen above, highlights the lesion within the LAD artery, marked with a red circle. This illustrates a mid-vessel filling defect suspicious of an isolated thrombus clot with further mild atheroma in the distal LAD. He was subsequently transferred for intracoronary OCT to assess the LAD lesion for further morphological clarification and to aid optimal management through percutaneous angioplasty. The OCT showed a ruptured plaque with some evidence of fresh red thrombus secondary to the endothelial damage, not exceeding 30% occlusion (see Figures 5-7).

We utilized the OCT standard protocol, with the acronym "MLD-MAX," to strategize our percutaneous coronary angioplasty. M.L.D is pre-PCI OCT, used to strategize the lesion, where "M" stands for morphology in search of calcium burden, "L" is the length to select landing zones based on healthy tissue/EEL visualization, "D" is the diameter to measure vessel, stent, and balloon diameters, M.A.X represents post-PCI OCT, aimed as optimization of stent deployment. M represents the medial dissection to be addressed,A for apposition to address gross malposition, and X expansion to confirm expansion.

Procedure summary

A PCI was performed on the proximal LAD artery using one drug-eluting stent (DES) under OCT guidance. An Extra Backup 3.5 catheter (Medtronic, Dublin, Ireland) and a Sion Blue wire (ASAHI-INTECC, USA) were used to access the LAD. The Sion Blue wire was subsequently advanced into the first diagonal branch (D1). OCT imaging revealed a plaque rupture with evidence of fresh red thrombus.

**Figure 8:**
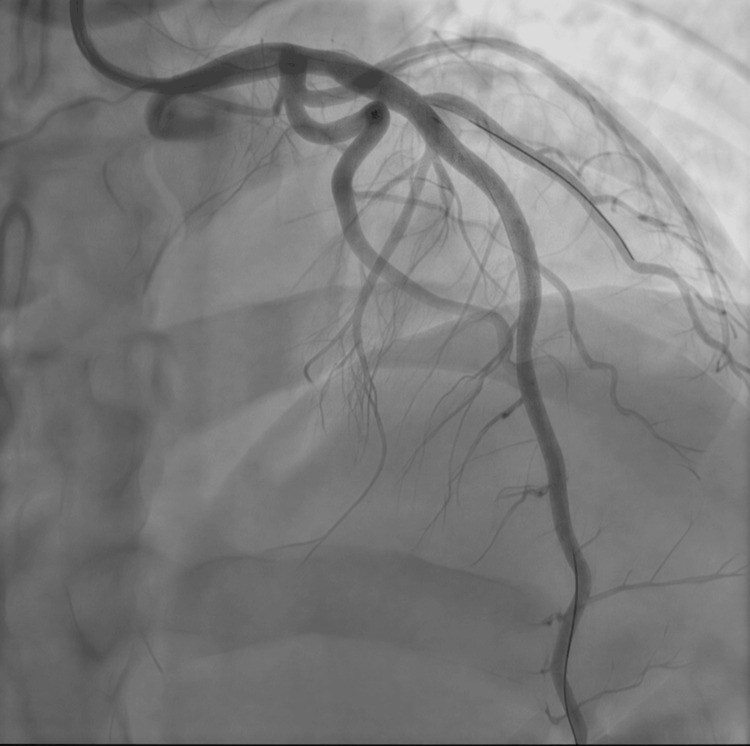
Angiography after stent insertion Post-stent dilatation shows excellent angiographic flow

The affected segment was directly stented across D1 using a 4.0 × 23 mm Xience DES (Abbott, Abbott Park, Illinois), deployed at 12 atm. The proximal optimization technique was then performed using a 4.5 × 12 mm noncompliant balloon. A follow-up OCT run demonstrated good stent apposition and expansion, with no evidence of stent edge dissection. The minimum stent area was 8 mm², and the minimum stent expansion was 94%. An excellent final angiographic result was achieved, and the procedure was completed successfully. Figure 8 shows the post-OCT-PCI angiogram image following stent placement.

Following a successful coronary angioplasty and stable condition 24 hours after the procedure, the patient was discharged on dual antiplatelet therapy (clopidogrel 75 mg daily and aspirin 75 mg daily) for one year, followed by lifelong single antiplatelet therapy (aspirin 75 mg daily). Cardiac rehabilitation and secondary prevention measures were advised, including optimal blood pressure control and high-dose statin therapy. His HbA1c was within normal range.

Omeprazole, a proton pump inhibitor, was prescribed to minimize the risk of dyspepsia or gastroduodenal ulceration. He was strongly advised to cease smoking and take alcohol only in moderation. On a follow-up three months later, the patient reported making a good recovery. He attended post-MI cardiac rehabilitation and is on smoking cessation therapy using nicotine replacement products. He reported that he had fully returned to his usual activities but was avoiding extreme exertional activities.

## Discussion

In this case, intracoronary OCT was utilized in conjunction with conventional coronary angiography. The incorporation of OCT significantly refined procedural assessment, lesion characterization, and optimization strategy compared with angiography alone. OCT provided high-resolution visualization of coronary microstructures not discernible with angiography or grayscale IVUS.

The 2023 European Society of Cardiology guidelines for the management of ACS recommend intravascular imaging to guide PCI in cases involving ambiguous culprit lesions [6]. Notably, some aspects of the guidelines have subsequently been corrected and updated [7]. These recommendations are supported by evidence from major clinical trials such as ILUMIEN IV, Optical Coherence Tomography Optimized Bifurcation Event Reduction, and OCTIVUS, which have demonstrated superior procedural optimization and improved clinical outcomes when PCI is guided by intracoronary imaging rather than angiography alone [8-10].

According to Jang et al., OCT identified plaque disruption in approximately 25% of patients with NSTE-ACS, a markedly higher prevalence than previously observed through angiographic evaluation [11]. In the present case, OCT clearly delineated the morphology of an angiographically ambiguous lesion in the LAD artery, excluding an isolated thrombus and revealing features consistent with plaque disruption as the underlying mechanism of ACS. Furthermore, OCT provided a detailed assessment of intimal and endothelial integrity, supporting plaque destabilization as the precipitating pathophysiological process.

OCT provides near-histologic axial resolution (10-20 µm), substantially exceeding that of IVUS (100-150 µm), enabling detailed visualization of coronary architecture, including thin fibrous caps, thrombus, and microstructural disruptions [12]. These advantages make OCT particularly useful for assessing stent strut apposition, neointimal coverage, and edge dissections-factors often underestimated or undetectable by angiography. While IVUS remains a valuable imaging modality, especially for evaluating deeper plaque components and heavily calcified lesions, OCT's superior resolution provides unmatched diagnostic accuracy for delineating stent-vessel interactions and lesion morphology [13].

Evidence from randomized controlled trials and registry data indicates that OCT guidance enhances intraprocedural detection of suboptimal stent deployment, influences real-time decision-making, and may improve clinical outcomes compared with angiography-guided PCI [8-9,14]. Consequently, the integration of OCT into PCI practice is increasingly advocated, particularly for complex or ambiguous cases where lesion morphology directly influences interventional strategy.

However, several challenges and limitations to routine OCT implementation persist. The technique requires contrast injection for blood clearance, which increases total contrast volume and may pose risks for patients with renal impairment [15]. Additionally, OCT's limited tissue penetration restricts its capacity to characterize deep calcium or lipid-rich plaques compared to IVUS. Technical constraints may also arise in tortuous or ostial lesions, as well as in hemodynamically unstable patients. Practical considerations, such as procedural costs, acquisition time, and operator training, continue to limit widespread adoption [16].

In summary, in this case of a 45-year-old man with minimal cardiovascular risk factors and an angiographically inconclusive LAD lesion, OCT-guided PCI provided diagnostic precision and procedural optimization unattainable with angiography alone. These findings underscore the expanding role of intracoronary imaging, particularly OCT, in improving the accuracy of PCI planning and execution. The broader adoption of OCT, particularly in high-volume, resource-rich centers, is warranted to enhance procedural success, stent performance, and long-term clinical outcomes.

## Conclusions

NSTE-ACS may present with a normal ECG and atypical chest symptoms, making diagnosis challenging and potentially leading to missed cases. In such scenarios, cardiac biomarkers, particularly troponin and a comprehensive clinical assessment incorporating cardiovascular risk factors, are critical for accurate diagnosis.

The utilization of intracoronary imaging modalities, such as IVUS and OCT, can provide valuable insights in cases with nonconventional ACS presentations and inconclusive angiographic findings, thereby facilitating more precise interventions and improving clinical outcomes.

However, the increased contrast dose required for OCT may exacerbate renal dysfunction. IVUS guidance, by contrast, offers high diagnostic utility with lower contrast requirements and is, therefore, preferable in patients with impaired renal function. Operators are, therefore, encouraged to integrate intracoronary imaging to guide their coronary interventional procedures where possible and appropriate.

## References

[1] Yonetsu T, Jang IK (2024). Cardiac optical coherence tomography: history, current status, and perspective. JACC Asia.

[2] Ali ZA, Shin D, Singh M (2024). Outcomes of coronary intravascular lithotripsy for the treatment of calcified nodules: a pooled analysis of the Disrupt CAD studies. EuroIntervention.

[3] Kumar A, Cannon CP (2009). Acute coronary syndromes: diagnosis and management, part I. Mayo Clin Proc.

[4] Raphael CE, Heit JA, Reeder GS, Bois MC, Maleszewski JJ, Tilbury RT, Holmes DR Jr (2018). Coronary embolus: an underappreciated cause of acute coronary syndromes. JACC Cardiovasc Interv.

[5] Gao X, Liu M, Xu J (2025). Impact of in-hospital oral beta-blockers initiation on long-term outcomes in ST-elevation myocardial infarction patients with cardiogenic shock. Front Med (Lausanne).

[6] Byrne RA, Rossello X, Coughlan JJ (2023). 2023 ESC Guidelines for the management of acute coronary syndromes: developed by the task force on the management of acute coronary syndromes of the European Society of Cardiology (ESC). Eur Heart J.

[7] (2024). Correction to: 2023 ESC Guidelines for the management of acute coronary syndromes: Developed by the task force on the management of acute coronary syndromes of the European Society of Cardiology (ESC). Eur Heart J.

[8] Ali ZA, Landmesser U, Maehara A (2024). OCT-guided vs angiography-guided coronary stent implantation in complex lesions: an ILUMIEN IV substudy. J Am Coll Cardiol.

[9] Holm NR, Andreasen LN, Neghabat O (2023). OCT or angiography guidance for PCI in complex bifurcation lesions. N Engl J Med.

[10] Yamaji K, Kanenawa K, Morofuji T (2024). Serial optical coherence tomography assessment of coronary atherosclerosis and long-term clinical outcomes. J Am Heart Assoc.

[11] Jang IK, Tearney GJ, MacNeill B (2005). In vivo characterization of coronary atherosclerotic plaque by use of optical coherence tomography. Circulation.

[12] Prati F, Regar E, Mintz GS (2010). Expert review document on methodology, terminology, and clinical applications of optical coherence tomography: physical principles, methodology of image acquisition, and clinical application for assessment of coronary arteries and atherosclerosis. Eur Heart J.

[13] Kubo T, Imanishi T, Takarada S (2007). Assessment of culprit lesion morphology in acute myocardial infarction: ability of optical coherence tomography compared with intravascular ultrasound and coronary angioscopy. J Am Coll Cardiol.

[14] Lee JM, Choi KH, Song YB (2023). Intravascular imaging-guided or angiography-guided complex PCI. N Engl J Med.

[15] Ahmed M, Javaid H, Talha Maniya M (2024). Optical coherence tomography-guided versus angiography-guided percutaneous coronary intervention: a meta-analysis of randomized controlled trials. Int J Cardiol Heart Vasc.

[16] Tearney GJ, Yabushita H, Houser SL (2003). Quantification of macrophage content in atherosclerotic plaques by optical coherence tomography. Circulation.

